# Effects of Dysesthesia-Matched Transcutaneous Electrical Nerve Stimulation Combined With Exercise Therapy on Tingling and Postural Stability in Cervical Spondylotic Myelopathy: A Case Report

**DOI:** 10.7759/cureus.111685

**Published:** 2026-06-28

**Authors:** Kotaro Kaito, Musashi Takagi, Yuki Nishi

**Affiliations:** 1 Department of Rehabilitation, Sendai Pain Clinic Center, Sendai, JPN; 2 Department of Rehabilitation, IMS Yokohama Higashi Totsuka General Rehabilitation Hospital, Yokohama, JPN; 3 Institute of Biomedical Sciences (Health Sciences), Nagasaki University, Nagasaki, JPN

**Keywords:** cervical spondylotic myelopathy (csm), dysesthesia, neuropathic pain treatment, postural stability, transcutaneous electrical nerve stimulation (tens)

## Abstract

To investigate the effects of an intervention combining transcutaneous electrical nerve stimulation (TENS), with parameters adjusted to match the patient's dysesthesia characteristics, and exercise therapy in a patient with cervical spondylotic myelopathy (CSM) who exhibited marked tingling of the lower limbs and impaired standing balance.

A 41-year-old woman diagnosed with CSM associated with spinal canal stenosis and disc herniation at the C5-C6 level underwent an intervention using a two-phase AB design consisting of an exercise therapy-only phase (Phase A) and a DM-TENS plus exercise therapy phase (Phase B). Phase A lasted seven days, and Phase B lasted eight days. On the first day of Phase B, the tingling and standing balance were evaluated as immediate changes in effect at three time points: before, immediately after, and 180 min post-intervention. Tingling and electric-shock pain were assessed daily using an 11-point numerical rating scale (NRS). Superficial plantar sensation and standing balance were assessed on the final day of each phase. The intervention consisted of a 60-min program combining TENS and exercise therapy. The effectiveness of TENS was analyzed using Tau-U statistics. Immediate improvements in tingling, electric-shock pain, superficial plantar sensation, and standing balance were observed following TENS; however, these symptoms worsened over time. After the intervention, improvements in tingling and electric-shock pain were observed as extended carry-over effects, along with maintained gains in superficial plantar sensation and standing balance. TENS, with stimulation parameters tailored to the characteristics of tingling, when combined with exercise therapy, may contribute not only to the relief of tingling and electric-shock pain but also to the improvement of motor performance.

## Introduction

Cervical spondylotic myelopathy (CSM) presents with a broad spectrum of symptoms, including motor and sensory dysfunctions in the upper and lower extremities, impaired standing balance and gait, and neuropathic pain [1]. Sensory disturbances, including numbness, tingling, and dysesthesia, are also common manifestations, with approximately 60% of patients experiencing numbness in the lower limbs [1].

Pharmacological agents are commonly used to manage neuropathic pain and sensory symptoms, including dysesthesia and numbness, in patients with CSM; however, their efficacy for neuropathic pain is modest, and adverse effects may be problematic [2]. In addition, pharmacological options specifically targeting sensory disturbances such as numbness remain understudied, prompting interest in non-pharmacological approaches. Recently, a novel transcutaneous electrical nerve stimulation (TENS) approach, termed dysesthesia-matched TENS (DM-TENS), which adjusts stimulation parameters based on an individual's abnormal sensory profile, has been reported to be effective in alleviating dysesthesia, including symptoms of numbness and tingling [3,4].

DM-TENS has been shown to improve not only dysesthesia and neuropathic pain but also motor behavior, as well as deficits in both static and dynamic sensory perception [3,5]. Because standing balance requires adequate sensory feedback, the improvement of dysesthesia through DM-TENS could lead to enhanced sensory clarity, which, in turn, may contribute to better standing balance. However, studies on the effects of DM-TENS on motor performance, particularly standing balance, remain limited.

Based on the above hypothesis, in this case report, we perform a detailed evaluation of the effects of DM-TENS on the sensory function and standing balance associated with improved tingling in a patient with CSM. Tingling, sensory function, and standing balance were measured repeatedly at short intervals to examine the effects of the DM-TENS and their interactions. Furthermore, the effects of combined exercise therapy and DM-TENS were evaluated to determine whether DM-TENS provided additional benefits to exercise therapy. Such a precise, individual-level analysis provides preliminary insights that may inform the design of future large-scale studies and optimization of intervention strategies.

## Case presentation

Case introduction

The patient was a 41-year-old woman. She initially experienced tingling in the left upper limb that progressively spread to all four limbs. She frequently dropped small objects and experienced frequent tripping while walking, which prompted her to consult her primary care physician. Magnetic resonance imaging revealed spinal canal stenosis at the C5-C6 level and a high T2 signal within the spinal cord, leading to the diagnosis of CSM with concurrent disc herniation.

Thirty-two days after diagnosis, she was admitted for surgery and underwent C4-C7 laminoplasty and C5-C6 anterior cervical discectomy and fusion. Sixteen days after surgery, the patient was transferred to our facility for intensive rehabilitation.

Her prescribed medications included domperidone (10 mg) three times daily, mirogabalin (5 mg) twice daily, tramadol hydrochloride (37.5 mg) four times daily, acetaminophen (325 mg) four times daily, and lubiprostone (24 μg) twice daily.

Tables 1-2 summarize the results of the neurological and proprioceptive assessments. Neurological evaluation revealed motor impairment, reduced superficial sensation below the C5 level, and impaired proprioception of the lower limb joints (hip, knee, and ankle).

**Table 1:** Neurological and proprioceptive assessments of patients. Muscle strength and superficial sensation (light touch and pinprick) in each spinal segment were assessed according to the American Spinal Injury Association (ASIA) International Standards for the Neurological Classification of Spinal Cord Injury. MMT is graded 0-5. ASIA sensory scores are graded as follows: 0 = absent, 1 = impaired, and 2 = normal. MMT: manual muscle testing; N/A: not available.

American Spinal Injury Association								
	MMT			Light touch			Pinprick	
	Right	Left		Right	Left		Right	Left
C2	N/A	N/A		2	2		2	2
C3	N/A	N/A		2	2		2	2
C4	N/A	N/A		2	2		2	2
C5	4	3		2	2		2	2
C6	4	4		1	2		1	2
C7	4	3		2	1		2	1
C8	4	4		2	1		2	1
T1	4	4		2	1		2	1
T2	N/A	N/A		2	2		2	2
T3	N/A	N/A		2	2		2	2
T4	N/A	N/A		2	2		2	2
T5	N/A	N/A		2	2		2	2
T6	N/A	N/A		2	2		2	2
T7	N/A	N/A		2	2		2	2
T8	N/A	N/A		2	2		2	2
T9	N/A	N/A		2	2		2	2
T10	N/A	N/A		1	1		1	1
T11	N/A	N/A		1	1		1	1
T12	N/A	N/A		1	1		1	1
L1	N/A	N/A		1	1		1	1
L2	4	4		1	1		1	1
L3	4	4		0	0		1	1
L4	4	4		0	0		1	0
L5	4	3		0	0		1	0
S1	3	3		0	0		0	0
S2	N/A	N/A		0	0		0	0
S3	N/A	N/A		0	0		0	0
S4-5	N/A	N/A		0	0		0	0
Total	39	36		36	34		39	35

**Table 2:** Results of the Joint Position Reproduction Test. The proprioception of the lower limb joints (hip, knee, and ankle) was evaluated using a Joint Position Reproduction Test; values represent the angular error (in degrees) between the target and reproduced joint positions, with larger values indicating greater proprioceptive impairment.

Joint Position Reproduction Test								
			Right			Left		
Hip			15°			26°		
Knee			9°			10°		
Ankle			8°			16°		

She reported spontaneous tingling and electric-shock pain in all four limbs, with pronounced symptoms in the distal regions, particularly in the hands, feet, and lower legs, where both sensations occurred simultaneously.

During her daily activities, she was at high risk of falling during standing tasks. Specifically, she was unable to maintain a standing posture independently when managing clothing in the restroom or when transferring to a wheelchair or requiring assistance. Because she required maximal assistance for walking, she used a wheelchair as her primary means of mobility.

This study was approved by the Ethics Committee of IMS Yokohama Higashi Totsuka General Rehabilitation Hospital. The patient provided written informed consent for publication of this report after receiving a detailed explanation of its purpose.

Intervention

Intervention Design

**Figure 1:**
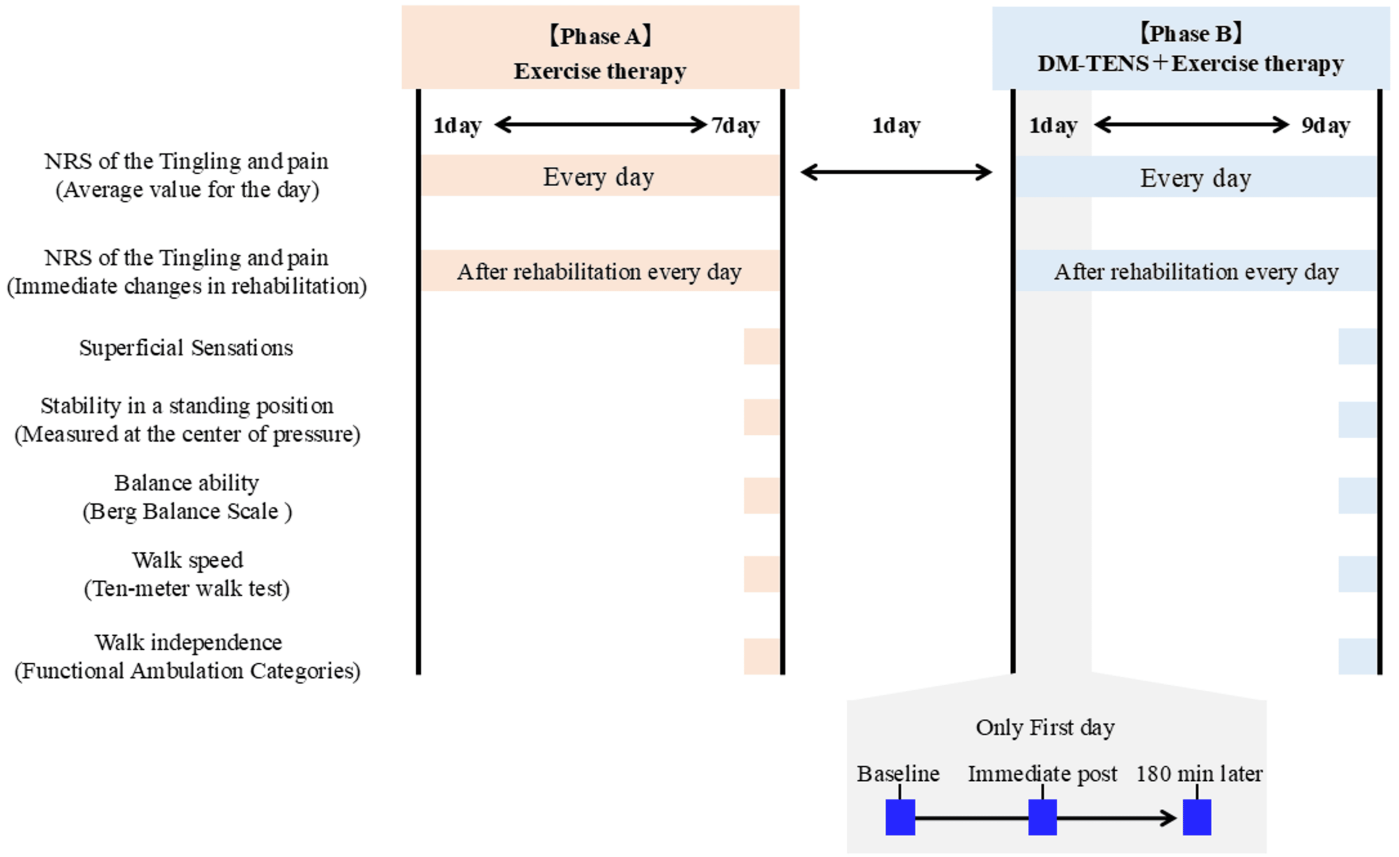
Intervention protocol used in this study. Phase A consisted of exercise therapy alone for seven consecutive days. Phase B involved the combined application of DM-TENS and exercise therapy over a total of nine days. Tingling and electric-shock pain were assessed daily as both the daily average and the post-treatment values. On the final day of each phase, motor function and superficial sensation were evaluated. In addition, on the first day of Phase B, immediate effects were evaluated at three time points: pre-intervention (baseline), immediately post-intervention (immediate post), and 180 min post-intervention (180 min later). This figure was created using Microsoft PowerPoint (Microsoft Corporation, Redmond, WA) and is not drawn to scale. DM-TENS: dysesthesia-matched transcutaneous electrical nerve stimulation.

Figure 1 illustrates the intervention design and timing of the outcome measurements used in this study. A two-phase AB design was adopted, consisting of an exercise therapy-only phase (Phase A) and a DM-TENS plus exercise therapy phase (Phase B), to evaluate the effects of combined DM-TENS and exercise therapy. In Phase A, only exercise therapy was provided, whereas in Phase B, the intervention consisted of a nine-day period combining DM-TENS and exercise therapy to assess sustained effects. On the first day of Phase B, tingling, standing balance, and superficial sensation were evaluated at three time points: before the intervention (baseline), immediately after the intervention (immediate post), and 180 min post-intervention (180 min later) to assess immediate and time-dependent effects. This structure enabled evaluation of changes observed across the two treatment phases.

Treatment Intervention

DM-TENS: DM-TENS stimulation (Espurge, Ito Physiotherapy, and Rehabilitation Co., Japan) used a continuous pulse pattern with a pulse width of 80 μs and a symmetrical biphasic waveform. Electrodes (self-adhesive, 5 × 5 cm; adhesive pad medium, Ito Physiotherapy and Rehabilitation Co., Japan) were applied to the L4 and L5 dermatomal areas of both lower limbs.

Stimulation parameters were based on those reported by Nishi et al. [5]. The timing and intensity of tingling were assessed through verbal confirmation with the patient during stimulation, during which the patient reported whether the electrical stimulation coincided with and resembled the perceived tingling in terms of frequency and intensity. Specifically, the stimulation frequency was adjusted to match the perceived frequency of tingling (i.e., the fineness of the tingling sensation), and the stimulation intensity was adjusted to match the perceived intensity of tingling. Through this individualized matching process, the stimulation frequency was determined to be 80 Hz. The stimulation intensity was adjusted within a range of 18-24 mA according to daily fluctuations in tingling intensity. During the adjustment, introspective feedback consistent with previous studies was confirmed, such as "the electrical stimulation coincides with and cancels out the tingling, resulting in clearer superficial sensation." For safety, the skin condition was inspected before and after each session to prevent skin complications caused by electrical stimulation.

Exercise therapy: In Phase A, exercise therapy was performed alone. In Phase B, the DM-TENS parameters were adjusted, and exercise therapy was administered while applying DM-TENS. Exercise therapy was administered for 60 min per session in both phases and included lower-limb stretching, manual resistance training targeting major lower-limb muscle groups, standing balance training, and gait training. The content of lower-limb stretching and resistance training was consistent between the two phases. The level of assistance required for standing balance and gait training was adjusted according to the patient's functional status during each session. In Phase A, standing balance and gait training were performed with substantial physical assistance and supportive devices, including a harness and a wheeled walker that enabled weight-bearing through the upper limbs. In Phase B, DM-TENS was applied throughout the session, and training was performed with minimal physical contact or without physical support; gait training was conducted with hand-held assistance or without physical support. Specific parameters, including repetition counts and gait distance, were adjusted dynamically according to the patient's fatigue and safety during each session rather than being predetermined.

Outcome Measures

Tingling and pain: Because the patient exhibited prominent tingling and electric-shock pain in the lower limbs, these symptoms were selected for assessment. Based on items from the Short-Form McGill Pain Questionnaire 2 (item 16: electric-shock pain; item 21: tingling or "pins and needles"), symptom intensity was assessed using an 11-point numerical rating scale (NRS) [6]. Both the daily average scores and the scores before and after each intervention were recorded.

Superficial plantar sensation: To evaluate the sensory changes associated with changes in tingling, the superficial plantar sensation was assessed. A standardized instrument was used for sensory evaluation (Semmes Weinstein Monofilament Set; SAKAI Medical Co., Japan). Evaluations were conducted on the final days of Phases A and B, as well as on the first day of Phase B to examine the immediate effects.

Standing stability: Standing stability was assessed by placing the patient on a force plate (Balance Wii Board; Nintendo Co., Japan), and the center of pressure (COP) under the feet was measured. The Balance Wii Board has been suggested as a tool with acceptable reliability and validity for assessing standing balance [7]. Measurements were obtained under eyes-open and eyes-closed conditions, with the patient instructed to gaze at a marker located 2 m ahead. Although a previous study on chronic stroke patients demonstrated the validity and reliability of 30-s recordings [8], the patient was unable to stand for more than 30 s; thus, all recordings were standardized to 10 s. The data were collected at a sampling frequency of 190 Hz, and the total COP path length and 95% confidence ellipse area were calculated [9]. MATLAB (version 2022b; MathWorks, Natick, Massachusetts) was used for data processing. Assessments were performed on the final days of Phases A and B and on the first day of Phase B to examine the immediate effects.

Overall balance ability: The Berg Balance Scale (BBS) was used to evaluate the overall balance ability. BBS assessments were conducted on the final days of Phases A and B and on the first day of Phase B to examine the immediate effects.

Gait ability based on walking speed and assistance level: The 10-m walking test and Functional Ambulation Categories (FAC) were used to assess changes in gait ability [10]. These assessments were also conducted on the final days of Phases A and B and on the first day of Phase B to examine the immediate effects.

Statistical analysis

To evaluate effect sizes for tingling and electric-shock pain, Tau-U analysis was performed separately for the pre- and post-intervention NRS data by comparing Phase A with Phase B [11]. Calculations were conducted using a web-based calculator (https://singlecaseresearch.org/calculators/tau-u/). Baseline correction was applied when a trend was observed, defined as a tau value exceeding 0.2. Tau-U values were interpreted as follows: ≤0.2 (small effect), 0.2-0.6 (moderate), 0.6-0.8 (large), and ≥0.8 (very large) [12]. Statistical significance was set at p < 0.05. Other outcomes, including COP measures, BBS, the 10-m walk test, and FAC, were analyzed descriptively.

Results

The patient reported no adverse effects while using the DM-TENS and was able to complete 60-min sessions. No burns or skin complications caused by the electrical stimulation were observed. Although Phase B was planned for nine days, the intervention was completed over eight days because of the patient's temporary illness. During Phase B, in which DM-TENS was combined with exercise therapy, both tingling and electric-shock pain were alleviated. No worsening of symptoms was observed during DM-TENS. While standing on the floor in a state in which the tingling had subsided, the patient offered the following introspection regarding the sensory and perceptual changes in the lower limbs during movement:

“Previously, I relied on the pain that appeared when I shifted my weight onto my feet to maintain balance. Now, it feels like I am standing on solid ground, like before the illness, and I can clearly perceive where my legs are.”

Although the intensity of the tingling varied over time and required frequent adjustments of the stimulation intensity, the frequency remained constant at 80 Hz.

Immediate and time-dependent changes in tingling and electric-shock pain

**Figure 2:**
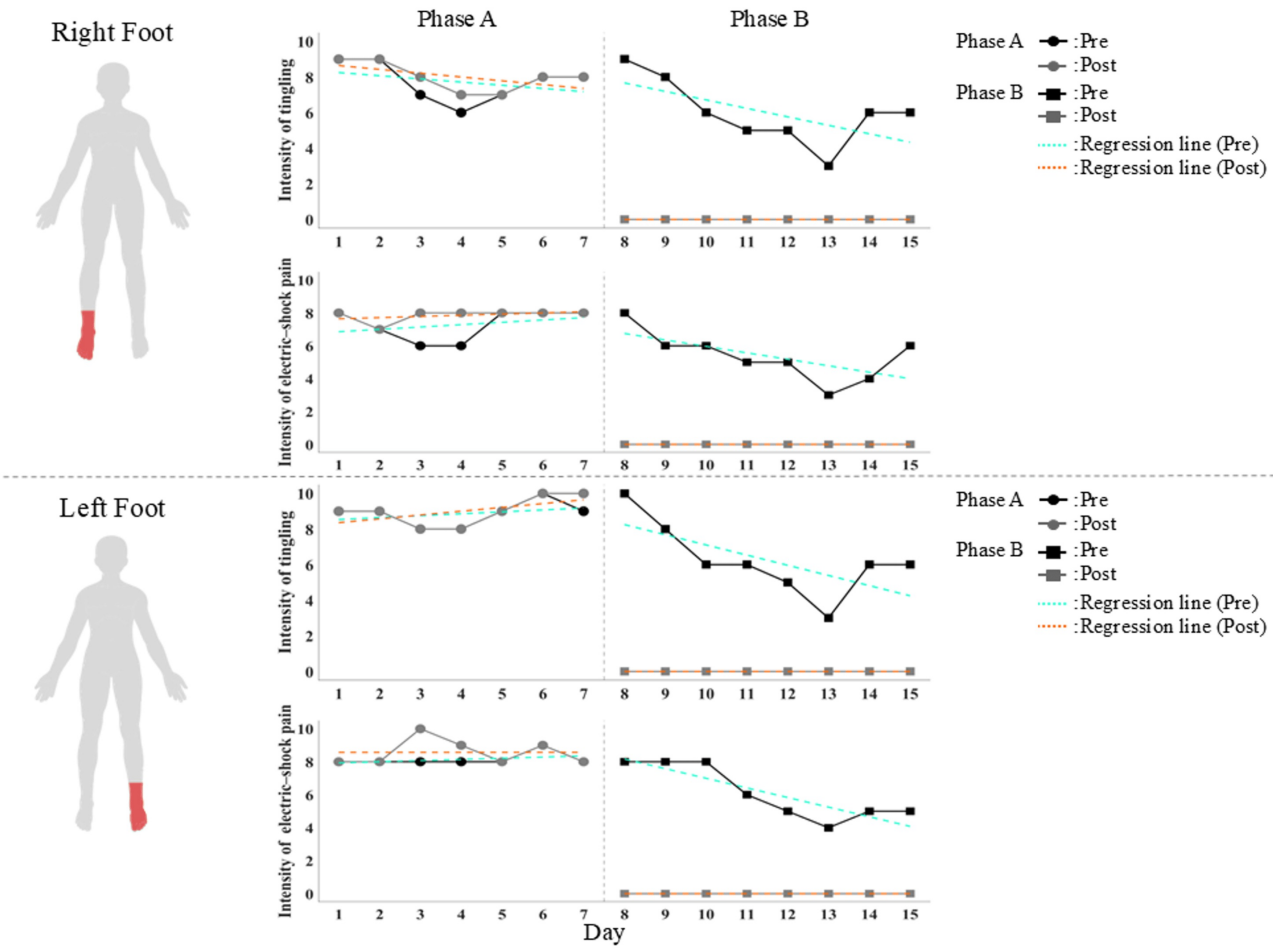
Changes in tingling and electric-shock pain. This graph illustrates the changes in tingling and electric-shock pain before and after treatment during each intervention period. Lower NRS scores indicate reduced symptom intensity, reflecting symptom improvement. NRS: numerical rating scale.

Figure 2 shows the pre- and post-intervention NRS scores for tingling and electric-shock pain across both phases, including between-phase differences in pre-intervention scores and short-term changes within each phase. In Phase A, immediate within-session changes were minimal, whereas notable immediate within-session improvements were observed in Phase B. Additionally, pre-intervention scores in Phase B were lower than those in Phase A, suggesting short-term carry-over effects during Phase B.

Mean pre-intervention NRS scores for tingling and electric-shock pain were lower in Phase B than in Phase A. Tingling in the right foot was 7.7 (± 1.1) in Phase A and 6.0 (± 1.9) in Phase B. In the left foot, scores were 8.9 (± 0.7) in Phase A and 6.3 (± 2.1) in Phase B. For electric-shock pain, the right foot showed 7.3 (± 1.0) in Phase A and 5.4 (± 1.5) in Phase B. The left foot showed 8.1 (± 0.4) in Phase A and 6.1 (± 1.6) in Phase B.

These differences were further supported by the Tau-U analysis, which indicated moderate-to-very large effects for both tingling and electric-shock pain. For tingling, the right foot showed a moderate preintervention effect, whereas the left foot showed a very large effect. After the intervention, large to very large effects were observed in both feet. For electric-shock pain, both feet consistently exhibited very large effects across the pre- and post-intervention comparisons (Table 3).

**Table 3:** Effect sizes from the Tau-U analysis for tingling and electric-shock pain. *p < 0.05, **p < 0.01. Negative Tau-U values indicate a decrease in NRS scores from Phase A to Phase B. Lower NRS scores indicate lower symptom intensity. CI: confidence interval; SD: standard deviation; VARs: variance; NRS: numerical rating scale.

		Baseline corrected	S	Tau	VARs	SD	Z	90% CI
Tingling								
Pre (Phase A vs. Phase B)	Right foot	Yes	–29	–0.55	298	17.3	–1.68^**^	(−1.00, −0.01)
	Left foot	Yes	–45	–0.83	298	17.3	–3.13^**^	(−1.00, −0.35)
Post (Phase A vs. Phase B)	Right foot	Yes	–48	–0.85	298	17.3	–3.00^**^	(−1.00, −0.30)
	Left foot	Yes	–56	–1.00	298	17.3	–3.13^**^	(−1.00, −0.64)
Electric-shock pain								
Pre (Phase A vs. Phase B)	Right foot	Yes	–44	–0.86	298	17.3	–2.55^*^	(−1.00, −0.28)
	Left foot	No	–56	–1.00	298	17.3	–3.24^**^	(−1.00, −0.49)
Post (Phase A vs. Phase B)	Right foot	Yes	–42	–0.89	298	17.3	–2.43^*^	(−1.00, −0.24)
	Left foot	No	–56	–1.00	298	17.3	–3.24^**^	(−1.00, −0.49)

Immediate changes in standing stability and superficial plantar sensation

**Figure 3:**
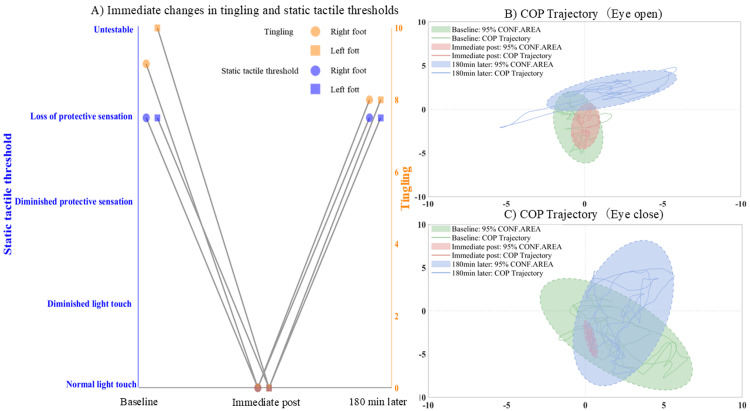
Time-series changes in tingling, superficial sensation, and COP trajectory observed on the first day of Phase B. (A) Degree of tingling and superficial sensory disturbance of the sole of the foot. (B) COP trajectory in the standing position (with eyes open). (C) COP trajectory in the standing position (with eyes closed). In panels (B and C), positive values on the Y-axis indicate forward, and positive values on the X-axis indicate right. A smaller confidence ellipse area and shorter COP path length indicate reduced postural sway. Data were processed using MATLAB (version 2022b; MathWorks, Natick, Massachusetts) and compiled into this figure using Microsoft PowerPoint (Microsoft Corporation, Redmond, Washington). CONF.AREA: 95% confidence ellipse area; COP: center of pressure; DM-TENS: dysesthesia-matched transcutaneous electrical nerve stimulation.

Figure 3 shows the changes in tingling, degree of superficial sensory disturbance, and COP trajectory at three time points: baseline, immediately post-intervention, and 180 min post-intervention. Video 1 illustrates the patient's standing balance and gait before the application of DM-TENS (corresponding to the "Baseline" condition in Figure 3) and during the stimulation period. Immediately after the intervention, the tingling resolved, superficial plantar sensation improved, and the standing balance stabilized. However, both tingling and superficial sensations deteriorated over time, returning to baseline levels after 180 min. The COP trajectory also showed changes in movement range, corresponding to time-dependent changes in tingling.

**Video 1:** Postural stability and gait performance associated with DM-TENS administration. Standing balance and gait performance before and during DM-TENS administration. DM-TENS: dysesthesia-matched transcutaneous electrical nerve stimulation.

Time-dependent changes in motor performance and superficial plantar sensation

Table 4 summarizes the results of motor performance and superficial plantar sensation across all evaluation points. Compared to the end of Phase A, motor performance showed an overall improvement at the end of Phase B, reaching levels equal to or better than those observed immediately after the initial intervention. However, the improvement in superficial plantar sensation was modest compared to the immediate post-intervention levels, with sensory impairment still present.

**Table 4:** Motor performance and superficial sensory perception of the sole at each evaluation point. For COP measures, lower values indicate reduced postural sway and improved standing stability. For BBS and FAC, higher scores indicate better performance. For the 10-m walk test, shorter times indicate faster gait speed. For superficial sensory grading, improvement is indicated by progression from LPS toward Normal LT. EO: eye open; EC: eye closed; Rt: right foot; Lt: left foot; LPS: loss of protective sensation; Normal LT: normal light touch; DPS: diminished protective sensation; COP: center of pressure; BBS: Berg Balance Scale; FAC: Functional Ambulation Categories.

		Phase A	Baseline	Immediate post	180 min later	Phase B
COP total track length (cm)	EO	47.9	69.2	26.9	91.3	23.3
	EC	90.7	81.9	31.2	99.8	31.9
95% confidence ellipse area (cm^2^)	EO	26.8	19.5	7.3	23.1	1.3
	EC	50.4	75.1	2.1	77.4	1.1
Berg Balance Scale		23	23	50	25	54
10-m walk test (sec)		25	28	16	38	11
Functional Ambulation Categories		1	1	3	1	4
Tingling	Rt	8	9	0	8	4
	Lt	8	10	0	8	4
Superficial sensory		LPS	LPS	Normal LT	LPS	DPS

## Discussion

To examine changes in tingling and motor performance associated with the addition of DM-TENS to exercise therapy, an AB single-case design was employed. The addition of DM-TENS to exercise therapy was associated with immediate improvements in tingling, electric-shock pain, and motor performance. Although relief from tingling was temporary, improvements in motor performance persisted, suggesting that exercise during transient tingling relief may help sustain functional gains.

The application of DM-TENS resulted in immediate relief from tingling, which is consistent with the findings of a previous study [3]. This immediate effect is thought to result from a busy-line effect, in which DM-TENS selectively blocks the sensory signals associated with paresthesia. In this case, the sustained effect was limited, with symptoms of tingling and pain that tended to re-emerge over time. Although the underlying factors contributing to the carry-over effects are not yet fully understood, patients with more severe sensory impairments may benefit less from DM-TENS [3]. In this case, the joint position sense in the lower limbs remained impaired upon re-evaluation, suggesting that the severity of sensory impairment may affect the sustainability of DM-TENS efficacy. Thus, future research should investigate the relationship between the degree of sensory impairment and the duration of therapeutic effects.

The temporal changes in tingling induced by the DM-TENS were paralleled by changes in the total path length and 95% confidence ellipse area of the COP trajectory. The total path length reflects the overall amount of sway, whereas the 95% confidence ellipse area represents the spatial extent of the sway. COP behavior partially reflects the maintenance of the center of mass within the base of the support and is associated with overall postural stability [13]. Concurrent reductions in both measures indicated that the COP oscillations were reduced and confined to a smaller area, suggesting improved postural stability. These findings suggest that the DM-TENS contributes to postural stability by alleviating tingling.

Postural control relies on the integration of multiple sensory modalities, including vision, vestibular input, and proprioception. A decline in sensory function is correlated with postural instability, as evidenced in individuals with cervical myelopathy [14] as well as healthy adults with reduced plantar sensitivity induced by cooling [15]. Therefore, securing sensory resources for postural control is essential. Nevertheless, individuals with sensory impairment may achieve postural stability by altering the weights of the remaining sensory modalities [16]. As presented in Figure 3, postural stability was found to be significantly reduced in the eyes-closed condition compared to the eyes-open condition. This indicates that the patient compensated for impaired proprioception and superficial sensations using visual input. When DM-TENS was applied, improvements in plantar superficial sensations were observed in parallel with reduced tingling, and this trend was synchronized with motor function improvements over time. We hypothesize that the masking effect of tingling on superficial sensation may have been alleviated by DM-TENS. Unlike tactile masking, in which concurrent sensory or pain stimuli increase tactile thresholds [17], the findings in this case raise the possibility that tingling itself may mask superficial sensation. It is therefore hypothesized that DM-TENS may have contributed to clarification of plantar superficial sensation by reducing tingling, thereby potentially enhancing the sensory resources available for postural control. This may have facilitated a shift in sensory weighting strategy, potentially contributing to the immediate improvement in postural stability observed in this case.

Although the enhancement of sensory weighting through increased sensory resources was associated with immediate postural stability, the patient's postural instability re-emerged on day 1 of Phase B as the tingling and sensory deficits returned. However, by the end of Phase B, postural stability was maintained despite the persistence of some degree of tingling and sensory impairment. This raises the possibility that mechanisms other than sensory weighting may be responsible for the carry-over effects. Motor learning typically involves the updating of internal models through error corrections based on sensory feedback [18]. Among the sensory modalities, proprioception plays a central role in motor learning. However, compensation using other modalities has been reported [19]. In addition, such adaptations may be generalized to movements performed without sensory feedback or to similar actions [20], implying that feedforward control can be achieved by updating the internal models based on the remaining sensory input. In the present study, repeated movements were performed during Phase B while applying DM-TENS, thus enhancing plantar sensation. This increase in sensory resources may have facilitated updating of internal models, potentially contributing to the development of feedforward control strategies adapted for postural stability. It is also hypothesized that, despite the persistence of tingling and sensory deficits, the patient maintained postural stability using feedforward strategies developed through sensory adaptation, although this hypothesis remains to be confirmed in future studies.

This study has four limitations. First, because postural instability involves multiple factors, the effects of DM-TENS should be interpreted in both the sensory and musculoskeletal contexts. Second, the 10-s COP measurement may have reduced data reliability. Third, the eight-day intervention limited our understanding of the long-term changes in tingling and motor performance. Fourth, several confounding factors, including recent spinal surgery and concurrent medications such as mirogabalin and tramadol hydrochloride, may have influenced the observed outcomes. Although the contribution of these factors cannot be ruled out, future studies should examine the effects of DM-TENS under controlled conditions to isolate its specific contribution to sensory and motor improvements.

## Conclusions

This case report explored the immediate and combined effects of DM-TENS and exercise therapy in a patient with CSM. DM-TENS was associated with immediate improvements in tingling, superficial sensation, and motor performance. When combined with exercise therapy, DM-TENS appeared to help maintain these improvements even with residual sensory deficits. These findings suggest that DM-TENS may enhance motor performance in patients with tingling and sensory impairments, although further studies are needed to confirm these preliminary results.
